# SWARMs Ontology: A Common Information Model for the Cooperation of Underwater Robots

**DOI:** 10.3390/s17030569

**Published:** 2017-03-11

**Authors:** Xin Li, Sonia Bilbao, Tamara Martín-Wanton, Joaquim Bastos, Jonathan Rodriguez

**Affiliations:** 1 Research Center on Software Technologies and Multimedia Systems for Sustainability (CITSEM), Technical University of Madrid, Ctra. Valencia, Km 7, 28031 Madrid, Spain; 2 TECNALIA, Parque Tecnológico de Bizkaia, C/Geldo, Edificio 700, 48160 Bizkaia, Spain; sonia.bilbao@tecnalia.com; 3 Hi-iberia Ingeniería y Proyectos, C/Juan Hurtado de Mendoza 14, 28036 Madrid, Spain; tmartin@hi-iberia.es; 4 Instituto de Telecomunicações, Campus Universitário de Santiago, 3810-193 Aveiro, Portugal; jbastos@av.it.pt; 5 Universidade de Aveiro, Campus Universitário de Santiago, 3810-193 Aveiro, Portugal; jonathan@ua.pt

**Keywords:** ontology, uncertainty, underwater robots, MEBN, knowledge representation

## Abstract

In order to facilitate cooperation between underwater robots, it is a must for robots to exchange information with unambiguous meaning. However, heterogeneity, existing in information pertaining to different robots, is a major obstruction. Therefore, this paper presents a networked ontology, named the Smart and Networking Underwater Robots in Cooperation Meshes (SWARMs) ontology, to address information heterogeneity and enable robots to have the same understanding of exchanged information. The SWARMs ontology uses a core ontology to interrelate a set of domain-specific ontologies, including the mission and planning, the robotic vehicle, the communication and networking, and the environment recognition and sensing ontology. In addition, the SWARMs ontology utilizes ontology constructs defined in the PR-OWL ontology to annotate context uncertainty based on the Multi-Entity Bayesian Network (MEBN) theory. Thus, the SWARMs ontology can provide both a formal specification for information that is necessarily exchanged between robots and a command and control entity, and also support for uncertainty reasoning. A scenario on chemical pollution monitoring is described and used to showcase how the SWARMs ontology can be instantiated, be extended, represent context uncertainty, and support uncertainty reasoning.

## 1. Introduction

Underwater robots are becoming more and more popular to carry out maritime operations, such as oil spill detection [1], bathymetric survey [2], plume tracking [3] and corrosion repair [4]. Underwater robots include heterogeneous vehicles capable of providing different functionalities, such as Autonomous Underwater Vehicles (AUVs), Autonomous Surface Vehicles (ASVs), and Remotely Operated Vehicles (ROVs), to perform maritime and underwater-related tasks. While dealing with complex missions that outmatch the capabilities of a single robotic vehicle (e.g., it is not realistic to use a single ROV for constructing a berm on seabed) or operations that are dangerous for divers, seamless cooperation between different vehicles is demanded to tackle the complexity or riskiness of maritime missions.

To enable cooperation between a variety of maritime robotic vehicles, namely produced by different manufacturers, thus opening up new applications and ensuring reusability, is one of the main objectives of the Smart and Networking Underwater Robots in Cooperation Meshes (SWARMs) (The SWARMs project: http://swarms.eu/) project, a European project in the field of underwater cyber-physical systems (CPS) and robotics. In order to achieve this ambitious goal, the SWARMs project must tackle a set of challenges [5], e.g., defiant maritime and underwater communication and data heterogeneity.

### 1.1. The Need for Semantic Interoperability in the Cooperation of Underwater Robots

In order to cooperatively carry out maritime operations, it is vital that vehicles should be able to collect information and share it among themselves, and with a command and control entity as well, without ambiguous meaning. A variety of information could be obtained from different data producers, such as sensors, vehicles, and external sources (e.g., weather forecast system). Substantial difficulties arise when attempting to exchange information between vehicles. In particular, data heterogeneity obstructs effective information exchange between vehicles to a great extent. Information, pertained to heterogeneous data producers, may be described in different formats, such as JavaScript Object Notation (JSON), depending on their type and involved manufacturer. In addition, even when different vehicles use the same terminology, sometimes it is interpreted with different meanings. For instance, the term Position is used to represent a local frame georeferenced location in robot A while robot B uses it to express its angular coordinates. Therefore, in order to tackle data heterogeneity, semantic interoperability must be guaranteed in the SWARMs system to enable transparent information sharing. It is a necessity to enable machine computable logic, inference, knowledge discovery, and data federation between computer systems (e.g., vehicles, and SWARMs platform components). Information exchanged must be explicitly modeled with rich semantics so that data heterogeneity can be abstracted and unambiguously understood by all involved robotic vehicles.

### 1.2. The SWARMs Approach

**Figure 1:**
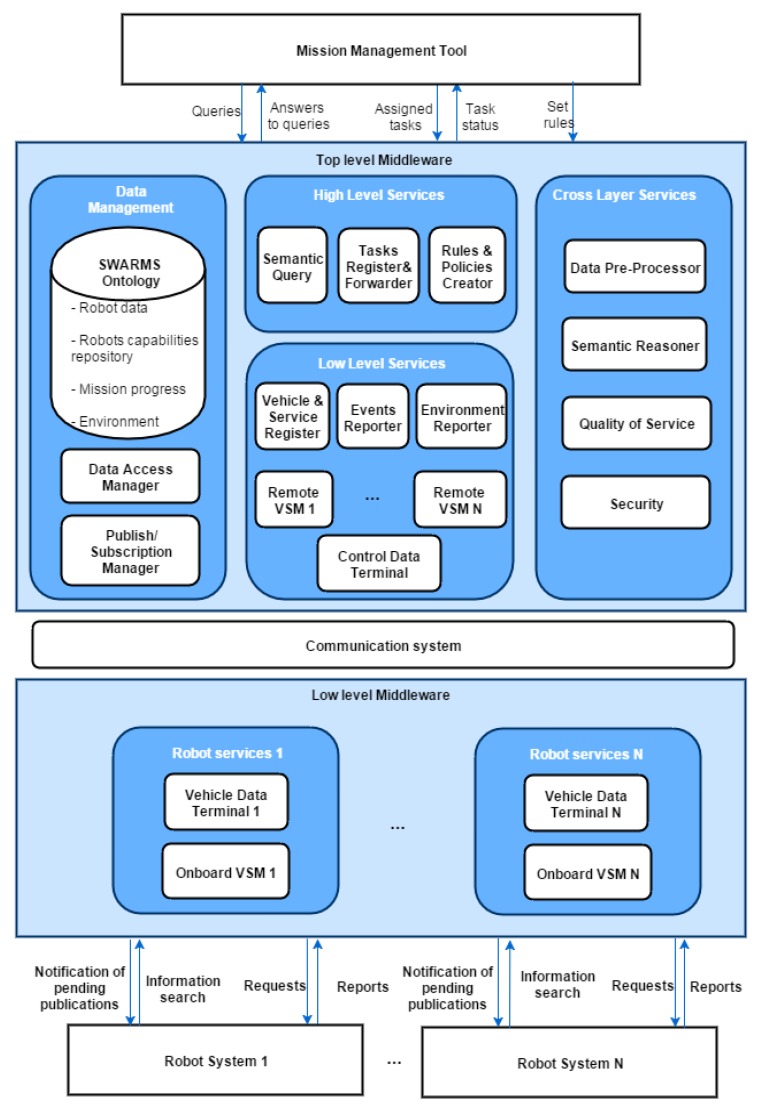
The SWARMs semantic middleware architecture.

Ontologies provide a formal specification of conceptualization in a well-defined and unambiguous manner; thus, they facilitate knowledge sharing and reusing. They are widely used to formally represent information in many domains, such as semantic web [6], smart home [7], and healthcare [8]. The SWARMs project also adopts ontologies to model all information that is necessarily exchanged between vehicles. A networked ontology is developed as a part of the SWARMs semantic middleware. As shown in Figure 1, the semantic middleware is defined to provide interactions between vehicles and the Mission Management Tool (MMT), which is located onshore or on a vessel and is responsible for the generation of missions, assignment of tasks to robots, and supervising the mission. More importantly, the semantic middleware can offer a variety of common services (e.g., security, context awareness, and publishing/subscription) for vehicles and MMT in the SWARMs project.

The networked ontology, encased in the SWARMS Ontology component, acts as a common information model to represent different domain-specific information and enables knowledge sharing within the SWARMs system. Specifically, it abstracts information, which is classified into four domains, including robotic vehicles, mission and planning, environment recognition and sensing, and communication and networking. In addition, it also provides valuable inputs for the context reasoner component to execute effective context reasoning. The context reasoner employs a hybrid context reasoning method [9], which includes ontological, rule-based, and Multi-Entity Bayesian Network (MEBN) reasoning. It is worth noting that information that is obtained by sensors or other sensing instruments is prone to be uncertain in the underwater environment [5] due to partial views, imperfect instruments, challenging communications, data loss, etc. Thus, uncertainty, as an inherent characteristic of information, also needs to be formally annotated in the SWARMs ontology for the sake of completeness in knowledge representation and also to provide support for uncertainty reasoning defined in the context reasoner component. The PR-OWL ontology, proposed by Rommel et al. [10], provides a principled formalism to represent uncertainty complying with the MEBN theory. It defines a set of ontology constructs that can be used to annotate probabilities about uncertain information and provide support for the hybrid context reasoning in the context reasoner. Thus, incorporated with PR-OWL ontology constructs, the SWARMs ontology can provide a complete and formal representation of information exchanged between vehicles, enable information sharing and reuse, and support for the ease of performing hybrid context reasoning.

### 1.3. Contributions Offered in This Paper

The main contributions provided by this manuscript can be summarized as follows:

- Thorough analysis of the domain and purpose of the SWARMs ontology and its requirements, including functional and non-functional requirements;
- Presentation of the SWARMs ontology, which is a network of different ontologies, along with brief descriptions of its main concepts and relationships; and
- Exploration of the applicability and extensibility of the SWARMs ontology by using a chemical pollution use case and a preliminary proof of its capability of supporting different context reasoning methods, including ontological, rule-based, and MEBN reasoning.

The remainder of this paper is structured as follows. Section 2 reviews existing ontologies in the field of cooperative underwater robots and off-the-shelf context reasoning for dealing with uncertainties. Afterward, Section 3 analyzes the main purpose and scope of the SWARMs ontology and requirements that it needs to meet. The SWARMs ontology is presented in Section 4. Then, a use case on chemical pollution detection is presented in Section 5 to showcase the applicability and extensibility of the SWARMs ontology and also verify its capability to support different context reasoning methods at a simulated level. Finally, Section 6 provides the conclusions and also points out future work.

## 2. Related Work

In this section, a review of existing ontologies relevant to the coordination and cooperation of underwater vehicles is presented. In addition, current solutions for addressing uncertainty in the knowledge representation and reasoning fields are reviewed.

### 2.1. Existing Ontologies for the Cooperation and Coordination of Underwater Robots

There is an upsurge in using ontologies to enable a formal representation for the robotic field [11]. The IEEE RAS Ontologies for Robotics and Automation Working Group [12] has been developing a standard ontology model to represent the knowledge and reasoning in autonomous robots, such as air, ground, and underwater vehicles. However, this ontology focuses on presenting a very high-level service representation for vehicles themselves, such as sensors, resources, capabilities, platform, tasks, and mission. Context, which is useful to characterize the operational environment of vehicles, is not considered by this ontology. The ontology proposed by Insaurralde et al. [13] also lacks the inclusion of environmental context, though it provides a good representation of planning and control systems for AUVs. The KnowRob [14] knowledge processing framework developed a set of ontologies to abstract robot actions, events, objects, environments, and the robot’s hardware as well as inference procedures that operate on this common representation. The KnowRob puts its emphasis on improving the autonomy of individual vehicles instead of enabling the cooperation and coordination of multiple vehicles. In addition, the household robots, rather than underwater robots, are the target modeling domain of the KnowRob ontologies. Similar to KnowRob, the ORO [15] system also leveraged a core robotics ontology to integrate data from diverse sources, such as sensors, domain knowledge, and human input. Nevertheless, the focus of the ORO ontologies is to help robots in interactions with humans. The RobotML [16] ontology, also known as PROTEUS ontology, was developed in the framework of the French research project PROTEUS [17]. The RobotML ontology aimed to enable scientific knowledge transfer between different robotic communities by formalizing the robots, their environments, robot parts, operations, mission, planning, their detailed behaviors, scenarios, etc. However, due to its complexity, the developed RobotML ontology cannot be directly used for exploitation as users must perform the semi-automatic transformation from the ontology to a UML representation. Besides, the RobotML is quite specific to their application; thus, it lacks generality to be reused in the underwater robots field. A reference ontology of collective behavior of autonomous was developed by Gorodetsky et al. [18]. This ontology provides model of the system and environment, model of interaction between the system objects and the environment, and model of the behavior of the system objects and the environment. Ontologies were also extensively adopted to provide semantic formalizations for many robotic applications, such as monitoring of the execution of robot plans [19], robots task planning [20], navigation planning [21], urban search and rescue missions with robots [22], space exploration using robots [23], and supervision of underwater environments for robots [24]. All these application specific ontologies for robots are conceived at a too specific and limited level to cover the overall modeling requirements from the cooperation of underwater vehicles. It is also worth noting that a common lack exists in all the aforementioned robotic ontologies, except the KnowRob framework. They assume a deterministic world without considering context uncertainties, let alone properly model them. In the underwater robots field, uncertainty is the norm rather than the exception in context data. Therefore, this paper will present an ontology for the representation of coordination and cooperation of underwater vehicles based on existing relevant robotic ontologies. The proposed SWARMs ontology is general enough to cover necessary context from different domains, such as mission and planning, communication and networking, robotic vehicle, and environment recognition and sensing. In addition, uncertainties, as an inherent characteristic of context data, will be modeled in the presented ontology. With the inclusion of uncertainties, the proposed SWARMs ontology can not only completely represent the world of knowledge but also provide the ease of using a reasoning system.

### 2.2. Existing Solutions for Modeling Uncertainties

The W3C UR3W-XG group [25] proposed an ontology of uncertainty which captures top-level classes and properties for characterizing the uncertainty consideration in ontologies. Uncertainty that might exist in ontologies is classified into five main types: *ambiguity*, *randomness*, *vagueness*, *inconsistency*, and *incompleteness*. Different mathematical theories could be used to deal with different types of uncertainty reasoning: fuzzy logic, Bayesian network (BN), Markov logic network, etc. In order to model different kinds of uncertainty and further provide support for uncertainty reasoning, several attempts have been followed. For instance, Bobillo et al. [26] introduced fuzzy logic in crisp ontologies to capture and represent vagueness of uncertain information. They proposed to model fuzzy ontology elements (e.g., fuzzy data types, fuzzy concepts, and fuzzy modifiers) by using the Web Ontology Language (OWL) 2 annotations. Probability is the best-known formalism for computational uncertainty reasoning, particularly effective for addressing randomness, inaccuracy, and incompleteness of uncertain information. While introducing uncertainty in knowledge representation languages has been an afterthought, it is common to use simple XML tags to express the probability value which is a number ranging from 0 to 1. However, this is just one simple aspect of probabilities. Probability has been regarded by many authors to be more about structure than it is about numbers. For instance, to model uncertainty from a probabilistic aspect and enable the ease of applying BN reasoning, Yang et al. [27] proposed a probability-annotation approach. Specifically, three ontology concepts, including “PriorProb”, “CondProb”, and “FullProbDist”, were defined to conceptualize collections of Bayesian related definitions. In addition, a data property “ProbValue” was proposed to link “PriorProb” and “CondProb” with the probabilistic value varying from 0 to 1. BN is effective to model and reason a fixed number of hypotheses. However, it cannot reason problems which involve a varying number of entities. To address this shortcoming of BN, Multi-Entity Bayesian Network (MEBN) [28], as an extension of standard BN, was proposed by Laskey et al. The PR-OWL ontology [29], which complies with the MEBN theory, was also proposed to annotate probability information about uncertain context. The MEBN could be very effective in reasoning problems in the presence of uncertainties with a varying number of entities involved. The approach of using the PR-OWL ontology to annotate uncertainty information in ontologies and provide support for MEBN reasoning has been adopted in many applications, such as procurement fraud detection [30], maritime awareness [31], and knowledge-driven analysis for cultural heritage [32]. As addressing context uncertainty, particularly focusing on its inaccuracy, incompleteness, and randomness features, this paper will firstly introduce the use of PR-OWL to annotate probability information and further provide support for MEBN reasoning over uncertainties in the underwater robot field. Incorporated with ontology constructs defined in the PR-OWL ontology, the proposed SWARMs ontology can properly model uncertainties and provide a complete representation for the coordination and cooperation of underwater vehicles. In addition, the probability-annotated SWARMs ontology can provide support for a hybrid context reasoning mechanism, including ontological, rule-based, and MEBN reasoning.

## 3. Modeling Considerations for the SWARMs Ontology

In this section, several modeling considerations are presented for the devisal of the SWARMs ontology. Specifically, Section 3.1 states the main purpose and scope of the SWARMs ontology. A set of functionalities that the SWARMs ontology can provide is discussed in Section 3.2.

### 3.1. Ontology Purpose and Scope

It is very important to clarify the domain and scope of the target modeling information. With a clear understanding of the purpose and scope, it is possible to define what concepts should be included or excluded from the SWARMs ontology. In addition, the viability, domain, and objectives of the SWARMs ontology can set requirements for the ontology design and provide an initial idea of the underlying semantics. The SWARMs ontology intends to model all information that is necessarily exchanged between any maritime or underwater vehicles and architecture components (e.g., middleware modules, MMT modules). In the scope of the SWARMs project and its application scenarios (e.g., oil spill detection, plume tracking, and berm construction), the wide range of information could be summarized into four different domains: robotic vehicles, mission and planning, environment recognition and sensing, and communication and networking. The purpose of developing the SWARMs ontology is to provide a formal representation of all four domain-specific information so that context heterogeneity can be abstracted and a common understanding can be achieved by vehicles and any SWARMs architecture components or entities. In addition, it is necessary to ensure extensibility of the SWARMs ontology so that it could be tailored to different scenarios with application extensions.

### 3.2. Ontology Requirements

In order to achieve the purpose and ensure an appropriate outcome, the SWARMs ontology should meet a set of requirements. These requirements can be grouped by non-functional and functional requirements.

#### 3.2.1. Non-Functional Requirements

Non-functional requirements are general requirements or aspects that an ontology should fulfill for the sake of modeling quality. They also refer to those ontological principles that guide the design process. According to the NeOn [33] methodology, *interoperability*, *modularity*, *reusability*, and *extensibility* are the important characteristics that an ontology should offer. Specifically, the SWARMs ontology should ensure interoperability between heterogeneous vehicles or software components in terms of syntax and semantics. With the interoperability feature, the SWARMs ontology will enable vehicles with a common understanding of the information exchanged between themselves. The SWARMs ontology should also be modular so that minimum coupling and maximum cohesion could be achieved. In addition, reusability is a must for the SWARMs ontology, thus supporting that its portions could be re-engineered in different domains or applications. The SWARMs ontology should be able to be extended with application enrichment. With the extensibility, it could be stretched in width in order to suit different applications or scenarios. In addition, the SWARMs ontology should be formalized following two more non-functional requirements [34]. (1) *Understandability*: The nomenclature of the SWARMs ontology should be easily understandable to all stakeholders, e.g., ontology engineers, marine experts, end users and operators. The naming for the SWARMs ontology elements should be self-explanatory and reveal an intuitive meaning; (2) *Conciseness*: To model the same domain of interest, a lightweight ontology is usually preferable to heavier ones. The SWARMs ontology should use the least number of words to express the most without redundancies so as to decrease the complexity of the design process.

#### 3.2.2. Functional Requirements

The SWARMs ontology can fulfill a set of functional requirements, namely, content-specific requirements, to fully represent information within the target scope. The functional requirements are grouped as follows.

- The SWARMs ontology must provide the mission and planning modeling. Two levels of abstraction of the mission and planning can be described by the SWARMs ontology. Firstly, the high-level planning that allows the user to describe different tasks regarding operations performed by a set of robotic vehicles without specifying the exact actions that each robotic vehicle needs to perform. The output of the high-level planning is a global mission plan consisting of the tasks that the swarm of robotic vehicles needs to perform. Secondly, low-level planning that is carried out at the robotic vehicle level and includes generation of waypoints, actions, and other similar low-level tasks. The output of the low-level planning is a vehicle plan. The mission and planning procedure needs to be decomposed and well represented in the SWARMs ontology so that vehicles can share tasks/operations/actions and understand them in the same manner so that cooperation and coordination could be fostered.
- The SWARMs ontology must provide a well-defined classification for the robots and vehicles that are used in the different missions and their attributes. Any information that could be useful for operators to understand the vehicles and their conditions is modeled in the SWARMs ontology. Different properties used to describe vehicles, such as motorized, propelled, non-motorized, speed, position, battery level, equipment, capabilities, and sensors onboard, are modeled with semantic annotations in the SWARMs ontology.
- The SWARMs ontology must provide an abstraction for communication and networking in the SWARMs architecture. It must describe the communication links available in SWARMs architecture to transfer information from the command and control station (CCS) to the vehicles and backward. In addition, it must provide modeling of the protocol and types of messages that can be transmitted within the SWARMs system.
- The SWARMs ontology must support the environment recognition and sensing modeling. Robotic vehicles involved in a mission should have a complete picture of the underwater environment so that they could better adapt to it accordingly. Thus, the SWARMs ontology provides a good representation of the underwater environment. Any information, that is defined targeting to characterize the environment, its recognition, and associated sensing, is properly modeled in the SWARMs ontology. For instance, sensors play a very significant role in sensing the environment and producing useful context data to represent it. The environment is defined through a set of main concepts, which are specified by particular properties that define the surroundings of the location where a mission or task takes place involving robotic vehicles. A variety of environmental properties, such as water salinity, conductivity, temperature, and currents, are formalized in the SWARMs ontology.
- The SWARMs ontology must model context uncertainties and support for uncertainty reasoning. The harsh maritime and underwater environment typically introduces uncertainties in context data, particularly in such data obtained by sensors or other sensing instruments. The SWARMs ontology can provide a suitable representation for context uncertainties for the sake of completeness and comprehensiveness. In addition, the uncertainty annotations provided by the SWARMs ontology are useful for further reasoning. In other words, the SWARMs ontology can enable the ease of applying uncertainty reasoning in order to generate more useful information.

## 4. The SWARMs Ontology

**Figure 2:**
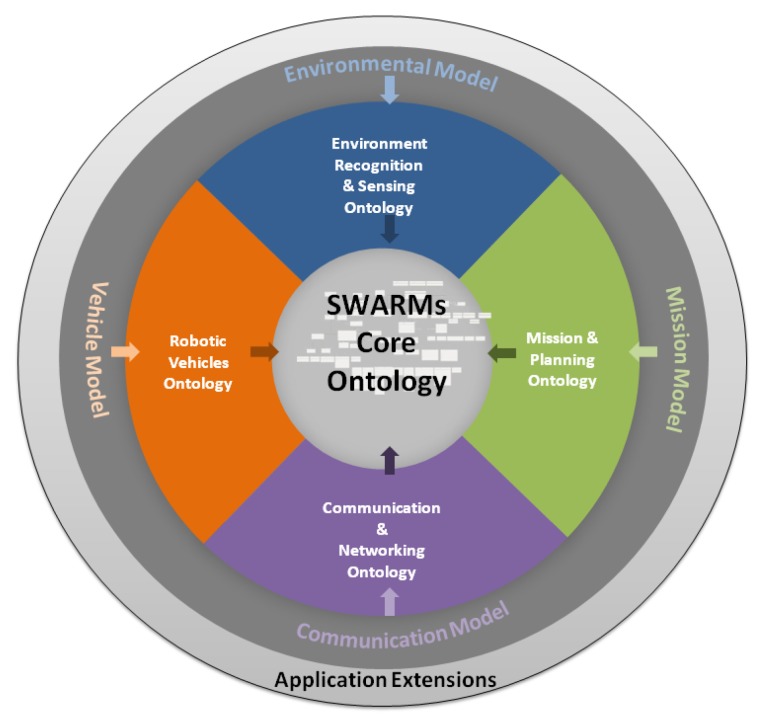
Overview of the SWARMs ontology.

To fulfill all the requirements presented in Section 3, the SWARMs ontology is designed as a network of ontologies. The overview of the structure of the SWARMs ontology can be seen in Figure 2. As depicted in Figure 2, the SWARMs ontology mainly consists of four domain-specific ontologies which model common concepts and aspects of certain domains, including mission and planning, environment recognition and sensing, robotic vehicle, and communication and networking. All these domain-specific ontologies are interlinked through a core ontology. In addition, the SWARMs ontology could be enriched with application extensions to suit certain use cases and scenarios. In the following sections, the specific design for different domain-specific ontology model and their relationships in the SWARMs ontology will be presented.

### 4.1. Core Ontology

**Figure 3:**
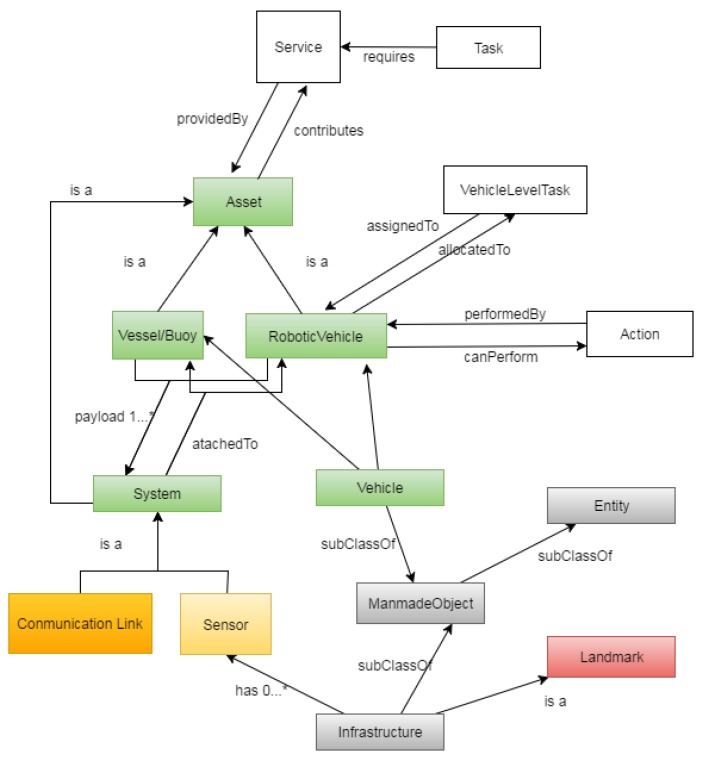
A representation of the overall structure of the core ontology.

The SWARMs ontology aims to model information mainly related to four domains. In order to link domain-specific ontologies and provide a coherent representation, a core ontology, shown in Figure 3, is presented. In Figure 3, the main concepts from the mission and planning ontology are depicted in white while concepts from the environment recognition and sensing domain are marked in grey, yellow, and red. In addition, ontology elements from the communication and networking domain and concepts from the robotic vehicles domain are displayed in orange and green, respectively.

As illustrated in Figure 3, services or capabilities (abstracted as the Concept Service in the mission and planning ontology) which are necessary to fulfill any task (modeled as the concept Task in the mission and planning ontology) are linked with Asset (defined in the robotic vehicles ontology) by using a pair of inversive relationships, namely, providedBy and contributes. The mission and planning ontology could also be interrelated with the robotic vehicles ontology at a lower abstraction level. Specifically, the concept VehicleLevelTask could be linked with RoboticVehicle by object properties (assignedTo and allocatedTo). In addition, a pair of inversive object properties, namely, performedBy and canPerform, are defined to describe the relationships between the concept Action and the concept RoboticVehicle. The concept Sensor from the environment recognition and sensing ontology and the concept CommunicationLink from the communication and networking ontology are modeled as subclasses of the concept System in the robotic vehicles ontology. The main concept Vehicle from the robotic vehicles ontology is subsumed into the concept ManmadeObject from the environment recognition and sensing ontology. With all the aforementioned ontology statements, the core ontology is able to glue different ontology elements from independent domain-specific domains and enable the building of a network of ontologies.

### 4.2. Robotic Vehicles Ontology

The Robotic Vehicles ontology models the robots and vehicles that are used in the different SWARMs missions (in the water domain only). Some important elements that capture the model are:

- Mobile robots are vehicles and robots (polymorphism);
- Robots are either autonomous robots, automated robots or remotely piloted robots (disjunction);
- Vehicles are either underwater vehicles or surface vehicles (disjunction); and
- Vehicles are either motorized or propelled by the environment/unmotorized (disjunction).

**Figure 4:**
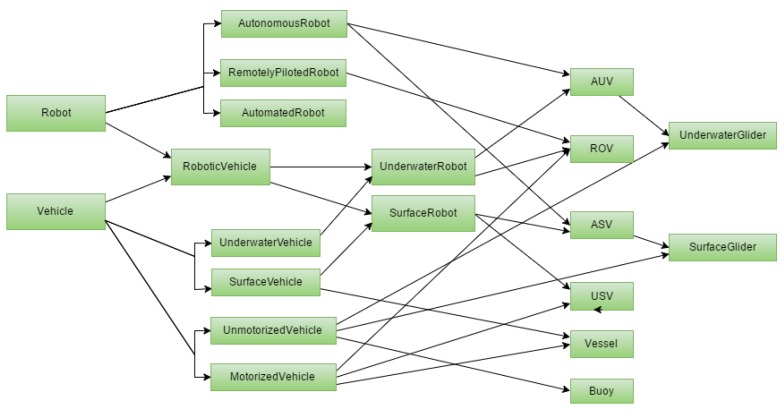
Robotic vehicle taxonomy.

Figure 4 shows the taxonomy of vehicles and robots used in SWARMs. A vehicle models any platform in or by which someone travels or something is carried or conveyed. Vehicles can travel underwater or operate on the surface of the water (e.g., Vessel). Motorized vehicles (e.g., ROV, USV, and Vessel) have propulsion systems on-board, unlike unmotorized vehicles (e.g., UnderwaterGlider, SurfaceGlider, and Buoy). A robot is a mechanical device that is capable of performing a variety of complex tasks on command or by being programmed in advance. It operates by remote control, automatically or autonomously. In a general view, this also includes humanoid and service robotics. A robotic vehicle is a physical object designed by a human agent to provide a service by acting here on the water domain. Remotely piloted robots (e.g., ROV) are robots that are piloted by one or several human operators that use sensory feedback. Automated robots are robots not remotely piloted (in their main role) that are able to act as an automation but not to adapt to changes in the environment and/or to follow scripted plans. Autonomous robots (e.g., AUV and ASV) are robots neither remotely piloted nor automated (in their main role) that are able to perform high level tasks and adapt to changes in the environment and operations with limited human intervention. Underwater robots (e.g., AUV and ROV) are underwater and robotic vehicles. Surface robots (e.g., ASV and USV) are surface and robotic vehicles. In this robotic vehicle domain-specific ontology, the UnderwaterGlider and SurfaceGlider can glide using density-volume changes without a propulsion system and they are subsumed into the AUV and ASV class, respectively.

### 4.3. Mission and Planning Ontology

**Figure 5:**
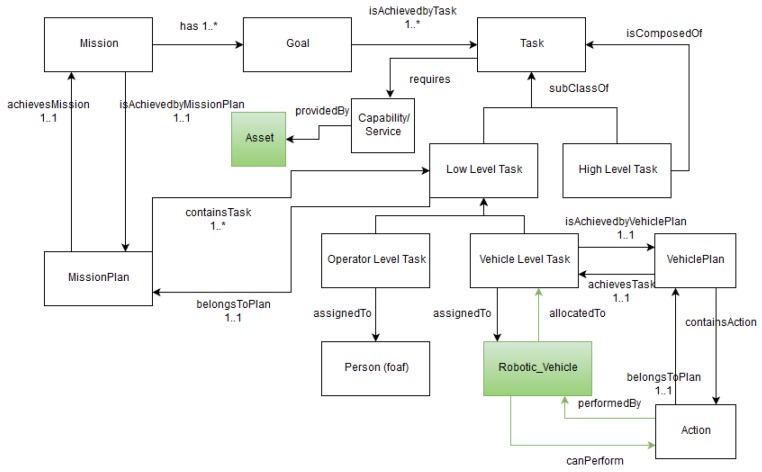
The mission and planning ontology.

The mission and planning ontology provides a general representation of the whole mission composition and planning procedure and of the low-level planning at a vehicle level as illustrated in Figure 5. A mission is defined as a set of goals to be performed by a swarm of vehicles (e.g., AUV, ROV, and USV) where each goal represents an objective to be achieved. Goals can be divided into subgoals. A goal is achieved by executing 1 to *n* tasks. These tasks can be of three types: operator level, vehicle level or high-level tasks. An operator level task is manually carried out by an operator. A vehicle level task can be carried out by one single vehicle (AUV, ROV or USV) whereas a high-level task is an assembly of tasks (operator level, vehicle level and/or high-level) that will be carried out by a swarm of vehicles. Tasks require capabilities to be performed (e.g., bathymetric sensors, H_2_S sensor or camera), a minimum battery level and have a start and end location. Vehicle level tasks lead to a set of actions (e.g., dive, go to waypoint, follow row or communicate status) to be performed by the vehicle.

Hence, a mission plan is abstracted as the sequence of scheduled low-level tasks (operator and vehicle level tasks) that need to be carried out by the swarm of vehicles to achieve a mission, with dependencies between tasks and approximate time duration. In addition, a vehicle plan is modeled as the sequence of actions that need to be carried out by a vehicle to achieve the set of tasks assigned to it in the global mission plan.

### 4.4. Environment Recognition and Sensing Ontology

This domain-specific ontology targets to characterize the environment, through recognition and sensing, where maritime or underwater missions will be carried out. The environment can be defined through a set of abstract concepts, which are specified by particular associated properties, that define the surroundings of the location where a particular mission or tasks take place involving robotic vehicles. This ontology is structured around a set of three disjoint spatial domain concepts, represented in blue, i.e., Surface, WaterColumn, and Seabed, each being characterized by multiple properties, some of which similar, e.g., Temperature and where Entities and Landmarks or Features can exist, and be found/recognized, or not, through sensing, in such SWARMs typical environment. Such concepts’ data properties, e.g., Temperature and most other sensed characteristics, must always be associated to a position and timestamp, which are typically provided by the vehicle that supplies the respective sensor(s) reading(s) in the characterization process.

Entities can be Biotic, i.e., animals or plants, or ManmadeObjects. Landmarks (or Features) represent all other kinds of objects that can be considered/recognized as landmarks, e.g., big rocks, as well as geological formations on the seabed. Infrastructures are considered usually as landmarks due to their size and construction records, i.e., are registered and mapped, and are also obviously manmade objects (polymorphism). Other objects, besides Infrastructures and objects with Sensors (e.g., AUV and ROV) are also considered, namely since it could be important in a mission or task to find/recognize them, e.g., oil pipes or components of an infrastructure.

**Figure 6:**
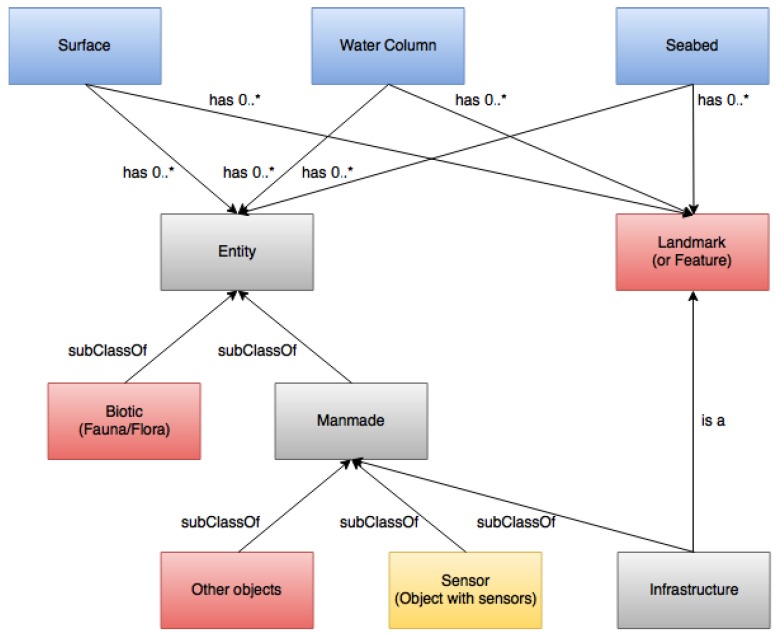
The environment recognition and sensing ontology.

Moreover, in the representation of the Environment recognition and sensing ontology in Figure 6, the concepts in red indicate the ones that could be important to be recognized after sensing, according to the specific SWARMs mission. In a comparable way, the Sensor concept is represented in yellow since it is the one that clearly is associated to the sensing part of the ontology, being the source of all sensing data, which is used to characterize the respective environment and allows the recognition of entities or objects and landmark or features. Typically, the Sensors are installed on robotic vehicles, but can also eventually be attached to other manmade objects, including Infrastructures. ManmadeObjects and Sensors are therefore evidently the interfacing concepts between this domain-specific ontology and the robotic vehicles ontology.

### 4.5. Communication and Networking Ontology

**Figure 7:**
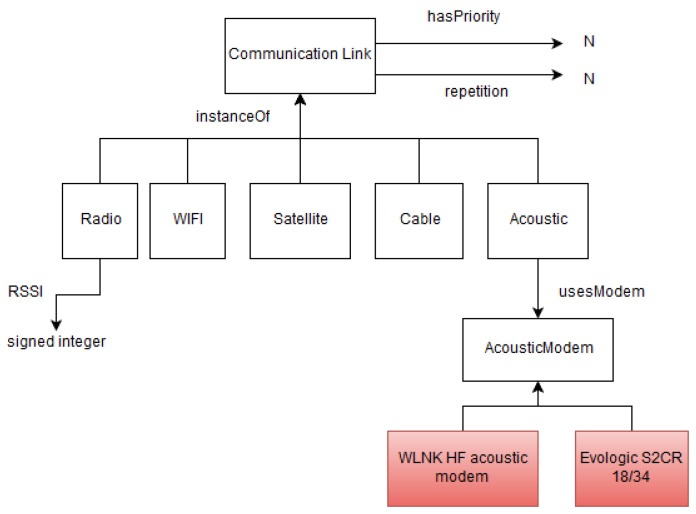
Communication and networking ontology.

This information model, shown in Figure 7, describes the communication links available in SWARMs to interconnect the different agents involved in a global mission execution and supervision, i.e., the ashore control station, support ships, USVs, AUVs, and ROVs.

The communication network should allow the data exchange in different environments (underwater and surface). The underwater environment is especially challenging as the propagation delay is significant, communication links are highly unstable and channel bandwidth is very limited.

In particular, the link between surfaced AUVs, USVs or buoys and the support ship is made through a radio frequency (RF) connection. In addition, satellite communication could be used with the CCS hosting the MMT, when offshore. For the underwater communication, acoustic modems are used with AUVs whereas ROVs are connected to the support ship by cables, which are used to transmit command and control signals between the operator and the ROVs.

### 4.6. Application Ontology

**Figure 8:**
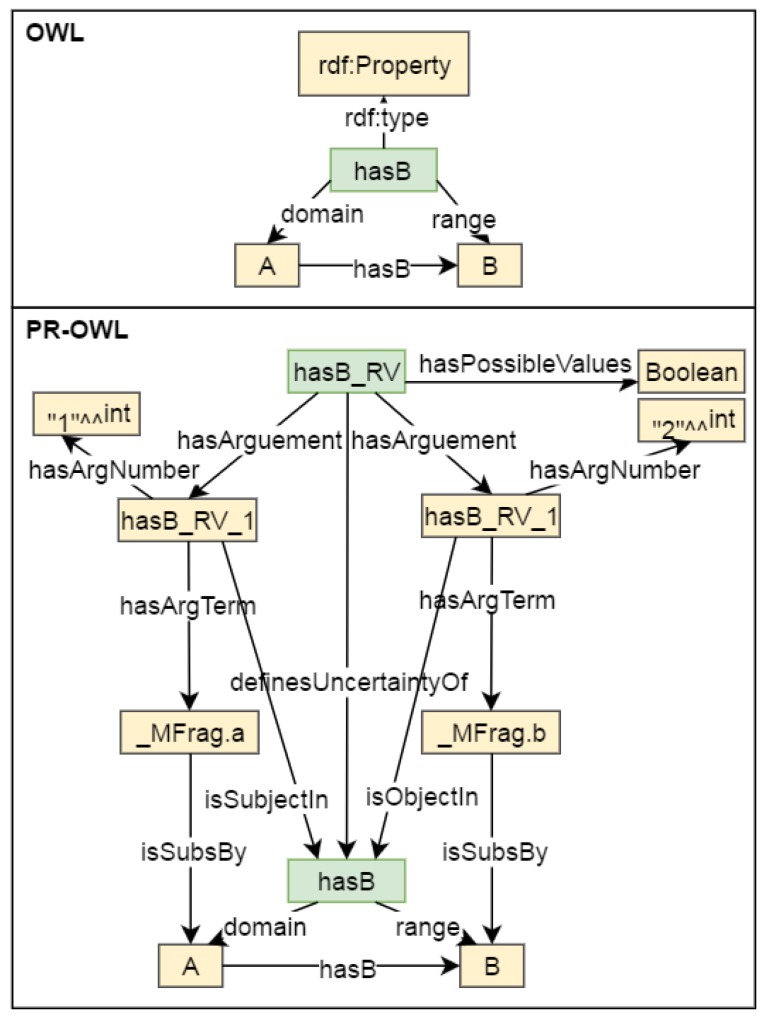
Mapping of PR-OWL random variables and OWL properties.

When dealing with different scenarios (e.g., oil spill detection, plume tracking, berm construction, and corrosion repair), the SWARMs ontology need be enriched with application extensions in order to accommodate application-specific requirements. In order to annotate uncertainties that are associated with context data in real applications, the PR-OWL ontology is inherited in the SWARMs ontology. The PR-OWL ontology provides a principled means to represent and reason about uncertainty. It goes beyond simply annotating ontologies with probabilities to provide a means of expressing subtle features required to express MEBN theories. A set of ontology constructs is defined to annotate uncertainty about attributes of and relationships among the entities compliant with MEBN theory. For instance, the mapping of PR-OWL random variables and OWL properties can be seen in Figure 8. The relationship between PR-OWL random variables and OWL properties is formalized using the relation *definesUncertaintyOf*. Besides, the relation *definesUncertaintyOf* can be used to relate the PR-OWL random variable *isObjectOfInterest* (Entity) to the OWL property *isObjectOfInterest*. Properties *isSubjectIn* and *isObjectIn* are defined to link arguments of the random variables with their OWL properties depending on whether they refer to the domain or range of the OWL property. The full specification for the PR-OWL ontology can be found in [10].

By providing a formal modeling of uncertainties in ontologies, the PR-OWL ontology could serve as a supporting tool for SWARMs applications that want to benefit from probabilistic inference within an ontology language. Incorporated with probabilistic extensions defined in PR-OWL, deterministic ontologies can be annotated with probability information about uncertain context and upgraded to probabilistic ontologies [10]. The development of the PR-OWL ontology and the formalization of it in OWL language can be enabled by the open source software tool UnBBayes (https://sourceforge.net/projects/unbbayes/). With the incorporation of PR-OWL, the SWARMs ontology can provide a comprehensive representation of specific applications in the presence of uncertainties. In addition, when attempting to represent a real application, the SWARMs ontology can be instantiated and provide support for the MEBN reasoning.

## 5. Implementation and Evaluation Using an Example

### 5.1. Formalization

**Figure 9:**
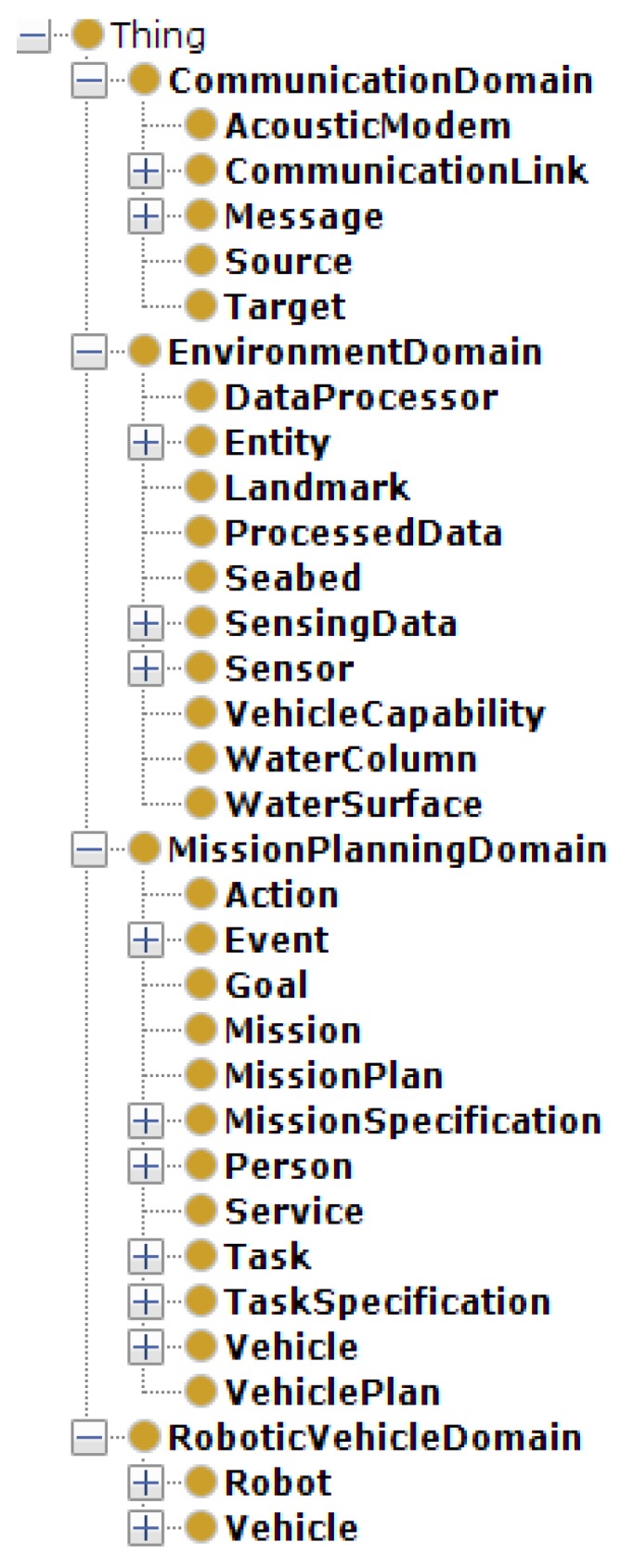
A snapshot of the hierarchy of the SWARMs ontology.

The proposed SWARMs ontology is developed using Protégé (http://protege.stanford.edu/) and formalized in OWL. The formalization of the SWARMs ontology in OWL allows a great level of expressivity while producing a model that can be easily shared through the web and thus be open to third party extensions. The proposed SWARMs ontology has been continuously inspected and evaluated by marine experts and ontology engineers along the development process. In addition, the reasoning result using the Pellet (https://www.w3.org/2001/sw/wiki/Pellet) reasoner has shown that the proposed SWARMs ontology is consistent. The hierarchy of the proposed SWARMs ontology is shown in Figure 9.

It is worth mentioning that the Semantic Web Rule Language (SWRL) (https://www.w3.org/Submission/SWRL/) is adopted in the SWARMs ontology to compensate the inability of OWL to express complex rule formations and relations. Essentially, SWRL rules are defined in an antecedent-and-consequent implication. By adding SWRL rules, the SWARMs ontology can be enhanced in terms of logicality, human-readability, expressivity, and completeness. Operators or marine experts can define rules through the MMT. The Rules and Policies Creator can translate those rules into the SWRL format and store them in the ontology model. Thus, the SWRL rules along with ontology instances in the ontology model can be important inputs for rule-based reasoning. Here, an example rule is provided by using the syntax of SWRL math built-ins.

Example rule:

WaterSurface(?x),hasWind(?x,?y),Wind(?y),WindSpeed(?y,?z),swrlb:greaterThan(?z,?20)->StrongWind(?y)

The rule above uses the SWRL syntax to express the definition of the concept StrongWind. It indicates that the wind speed, above the investigated water surface, which is faster than 20 m/s, can be classified as strong wind. Based on this SWRL rule, the rule-based reasoning can deduce if the wind above the water surface can be regarded as strong or not.

### 5.2. A Use Case on Chemical Pollution Monitoring

In this section, the proposed SWARMs ontology will be verified by using a chemical pollution monitoring scenario in terms of its applicability and extensibility. In addition, its capability of annotating uncertain information and supporting MEBN reasoning will be shown through this case study.

#### 5.2.1. Description of the Scenario

Chemical pollution inspection is a significant use case defined in the SWARMs project. The effective inspection could be very useful mainly in three situations, including corrective/predictive chemical pollution inspection, after an extreme condition weather, or after an earthquake. For instance, after an earthquake, marine biologists observe some dead fishes on the beach. They suspect that the Hydrogen Sulfide (H_2_S) in a given area of sea (*rgn1*) is the cause. A mission (*mission1*: *inspect the water column rgn1*) is given to the MMT. Based on available vehicles and their capabilities, the MMT generates a mission plan (*mp1*) in order to achieve the goals (e.g., *goal1: pollution is detected*, *goal2: emergency level of pollution is estimated*) of the *mission1*. The *mp1* is a plan which involves two AUVs (*auv1* and *auv2*) with different capabilities (e.g., *take videos and measurement of the concentration of H_2_S*) to accomplish the mission. Equipped with sensors such as *H_2_S probe*, *underwater camera*, *lighting system* and *side-scan sonar*, the two AUVs collaboratively detect H_2_S at two different depths (*p1* and *p2*). In addition, a set of context information, such as weather (is clement in *rgn1*), windspeed (is fast in *rgn1*), and different concentration level (*H_2_S* in *p1* is thin while *H_2_S* in *p2* is dense) are also obtained. Based on available context data, marine biologists want to know the emergency level of the polluted region so that they can estimate the biological impact of H_2_S and further take remedial measures accordingly.

#### 5.2.2. Ontology Extensions for the Scenario

To represent the specific scenario described in Section 5.2.1, some ontology elements defined in the SWARMs ontology need to be instantiated. Besides, additional ontology extensions, such as concepts and object properties, need to be added in order to provide a complete modeling for the scenario. Specifically, The *mission1: inspect the water column rgn1* is an individual of concept Mission. Its goals (e.g., *goal1* and *goal2*) can be inserted as instances of concept Goal. The mission plan *mp1* generated by the MMT is an instance of concept Mission. The *mp1* would include a set of tasks which could be created as instances of concept Task and refined as instances of concept LowLevelTask or HighLevelTask depending on their level. Vehicles involved in this mission, *auv1* and *auv2*, are individuals of concept AUV defined in the robotic vehicle domain-specific ontology. Different capabilities provided by vehicles, such as *move*, *take videos*, *measure the concentration of H_2_S*, can be instances of concept Service and be linked to concept AUV through an object property *providedBy*. Sensors like *H_2_S probe*, *underwater camera with lighting system*, and *side-scan sonar*, are created as instances of concept Sensor. The detected chemical material, H_2_S, can be populated as an instance of concept Pollutant (as a sub-class of other objects concept) within the environment recognition and sensing domain-specific ontology.

**Figure 10:**
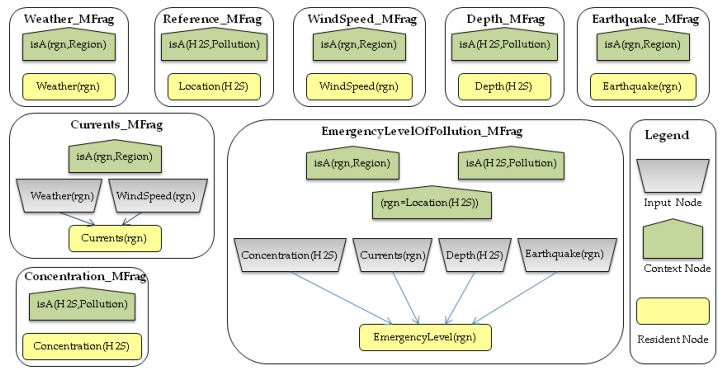
The probabilistic ontology for estimating emergency level of pollution in an MEBN theory.

It is worth noting that some environmental context data, useful to describe this scenario, including, weather, depth, concentration level, and spread speed, need to be captured and modeled into the SWARMs ontology in order to fully meet the application-specific modeling requirements. In addition, uncertainty, inherent to instances of context data, should also be modeled. As mentioned previously, the MEBN theory is adopted to model and reason about context uncertainty. Therefore, in order to model the application-specific information, an MEBN model, shown in Figure 10, is created using the UnBBayes tool and formalized in a probabilistic ontology (https://archive.org/download/purl_xinli) using PR-OWL constructs.

**Figure 11:**
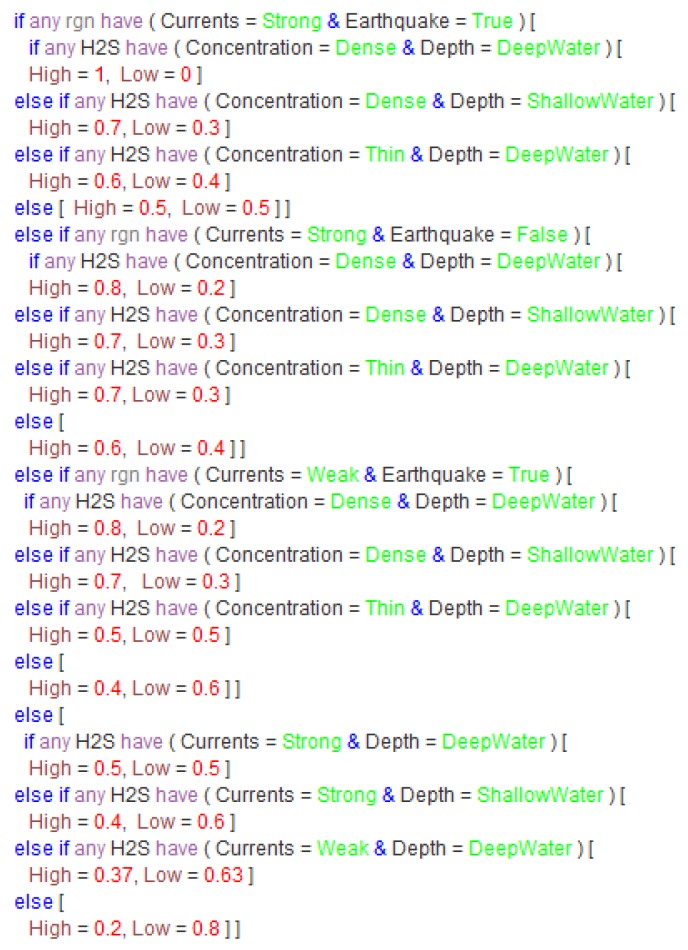
The joint probability distribution of emergency level.

**Figure 12:**
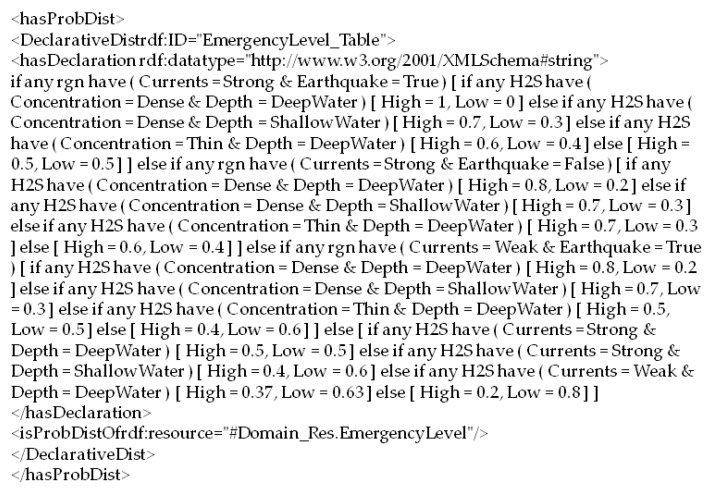
The OWL annotation for the emergency level probability distribution.

The probabilistic ontology, shown in Figure 10, models that the weather and wind speed in the investigated sea region can affect the currents in the same region. Currents, along with the concentration of H_2_S, depth of H_2_S, and earthquake occurrence in the sea region, are the influential factors to estimate the emergency level of the polluted sea region. The probabilistic ontology uses ontology constructs defined in the PR-OWL ontology to annotate probability information of those factors. For instance, the joint probability distribution of emergency level, shown in Figure 11, can be annotated using OWL constructs shown in Figure 12. Specifically, the probability distribution of EmergencyLevel is annotated as a string by using a *hasDeclaration* datatype. For the demonstration purpose, the probability information shown in Figure 11 is directly provided by marine experts.

Incorporating with the probabilistic enrichments, the SWARMs ontology is able to accommodate new application requirements and provide a comprehensive representation of this scenario and also its context uncertainty.

#### 5.2.3. MEBN Reasoning for the Scenario

**Figure 13:**
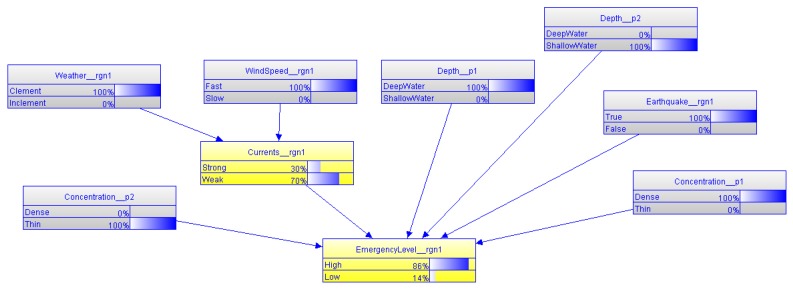
The MEBN reasoning for the emergency level.

Beyond the capability of representing the specific scenario, the SWARMs ontology could also provide support for the MEBN reasoning in order to deduce the emergency level of the polluted sea region. The information encased in the SWARMs ontology could serve as valuable inputs for the MEBN reasoning. With information, including an earthquake occurred in the sea region, the weather above the sea region is clement, the wind speed is fast, H_2_S pollution 1 is found in deep water, H_2_S pollution 2 is found in shallow water, the concentration of pollution 1 is dense, and the concentration of pollution 2 is thin, the MEBN reasoning, shown in Figure 13, can infer that the pollution in the inspected sea region *rgn1* is quite serious with a probability of 86%. Based on the reasoning result, marine biologists can have a more direct impression on the severity of the situation.

## 6. Conclusions

To enable heterogeneous underwater robots with a common understanding of information that is necessarily exchanged between themselves is a challenge in the cooperation of underwater robots. The SWARMs project is a European project that targets to tackle this challenge. The approach defined in the SWARMs project has been presented in this paper along with preliminary results. Specifically, a networked ontology, namely the SWARMs ontology, which is defined within the scope of the SWARMs project, has been introduced in this paper. The SWARMs ontology consists of several domain-specific ontologies, which are interrelated by a core ontology. Specifically, information, related to the mission and planning, the robotic vehicles, the environment recognition and sensing, and the communication and network domains, has been abstracted in the SWARMs ontology. In addition, uncertainty, as an inherent characteristic of information obtained in the harsh maritime and underwater environment, has been modeled in the SWARMs ontology by using ontology constructs defined in the PR-OWL ontology. The uncertainty modeling in the SWARMs ontology has followed the MEBN theory. Thus, the SWARMs ontology can provide both a comprehensive representation of information and support for a hybrid context reasoning, including ontological, rule-based, and uncertainty reasoning. Underwater robots and CCS/MMT can exchange information without ambiguous meaning if they comply with the SWARMs ontology. In this way, further cooperation and coordination of a group of vehicles could be potentially achieved. A chemical pollution scenario has been described and it has been used to showcase how the SWARMS ontology can be instantiated, be extended with application enrichments, represent context uncertainty based on the MEBN theory, and provide support for the MEBN reasoning.

Future work could be focused on the following aspects.

The SWARMs ontology should be tested with more use cases (e.g., berm construction, plume tracking) defined in the scope of the SWARMs project in terms of its applicability and extensibility.

Currently, the SWARMs ontology has been fully implemented. However, it has not been integrated into the ontology model component defined in the SWARMs middleware architecture. To develop it in the middleware architecture and ensure its interactions with other middleware components are also intended as the future work. With the complete implementation, evaluation of the SWARMs ontology can be more convincible.

Although the SWARMs ontology is designed to provide a common information model for the cooperation of underwater vehicles in the scope of the SWARMs project, the possibility of reusing it or its portions in other underwater robotics projects is worth to be explored.

The SWARMs ontology has inherited ontology constructs defined in the PR-OWL ontology to annotate context uncertainties based on MEBN theories. In this way, three aspects of context uncertainty, namely, inaccuracy, randomness, and incompleteness, have been resolved. However, other aspects of context uncertainty, such as vagueness and impreciseness, also need to be tackled. Introducing fuzzy logic into the SWARMs ontology to represent the vagueness and impreciseness of information will also be done in the future.
